# *Ascaris lumbricoides* β carbonic anhydrase: a potential target enzyme for treatment of ascariasis

**DOI:** 10.1186/s13071-015-1098-5

**Published:** 2015-09-18

**Authors:** Reza Zolfaghari Emameh, Marianne Kuuslahti, Daniela Vullo, Harlan R. Barker, Claudiu T. Supuran, Seppo Parkkila

**Affiliations:** Department of Anatomy, School of Medicine, University of Tampere, Tampere, Finland; BioMediTech, University of Tampere, Tampere, Finland; Fimlab Laboratories Ltd and Tampere University Hospital, Tampere, Finland; Dipartimento di Chimica, Laboratorio di Chimica Bioinorganica, Universita’ degli Studi di Firenze, Sesto Fiorentino, Firenze Italy; Neurofarba Department, Sezione di Scienze Farmaceutiche e Nutraceutiche, Universita’ degli Studi di Firenze, Sesto Fiorentino, Firenze Italy

**Keywords:** *Ascaris lumbricoides*, Beta carbonic anhydrase, Enzyme inhibition, Sulfonamide, Acetazolamide, Bioinformatics, Computational biology, Ascariasis

## Abstract

**Background:**

A parasitic roundworm, *Ascaris lumbricoides*, is the causative agent of ascariasis, with approximately 760 million cases around the world. Helminthic infections occur with a high prevalence mostly in tropical and developing xcountries. Therefore, design of affordable broad-spectrum anti-helminthic agents against a variety of pathogens, including not only *A. lumbricoides* but also hookworms and whipworms, is desirable. Beta carbonic anhydrases (β-CAs) are considered promising targets of novel anthelminthics because these enzymes are present in various parasites, while completely absent in vertebrates.

**Methods:**

In this study, we identified an *A. lumbricoides* β-CA (AIBCA) protein from protein sequence data using bioinformatics tools. We used computational biology resources and methods (including InterPro, CATH/Gene3D, KEGG, and METACYC) to analyze AlBCA and define potential roles of this enzyme in biological pathways. The *AlBCA* gene was cloned into pFastBac1, and recombinant AIBCA was produced in sf-9 insect cells. Kinetics of AlBCA were analyzed by a stopped-flow method.

**Results:**

Multiple sequence alignment revealed that AIBCA contains the two sequence motifs, CXDXR and HXXC, typical for β-CAs. Recombinant AIBCA showed significant CA catalytic activity with k_cat_ of 6.0 × 10^5^ s^−1^ and k_cat_/K_M_ of 4.3 × 10^7^ M^−1^ s^−1^. The classical CA inhibitor, acetazolamide, showed an inhibition constant of 84.1 nM. Computational modeling suggests that the molecular architecture of AIBCA is highly similar to several other known β-CA structures. Functional predictions suggest that AIBCA might play a role in bicarbonate-mediated metabolic pathways, such as gluconeogenesis and removal of metabolically produced cyanate.

**Conclusions:**

These results open new avenues to further investigate the precise functions of β-CAs in parasites and suggest that novel β-CA specific inhibitors should be developed and tested against helminthic diseases.

**Electronic supplementary material:**

The online version of this article (doi:10.1186/s13071-015-1098-5) contains supplementary material, which is available to authorized users.

## Background

Two parasitic worms, *Ascaris lumbricoides* and *Ascaris suum* were independently nominated by Linnaeus in 1758 and Goeze in 1782, respectively. Recent genetic and paleoparasitological evidence has suggested that these strains are, in fact, a single species [1]. Therefore, the original name, *A. lumbricoides*, should be used upon priority on taxonomic nomination.

Around 760 million people worldwide are infected with *A. lumbricoides*, mainly in Southeast Asia [2]. The human ascariasis infection is normally caused by feces contamination in water, vegetables, and other food. The eggs of the worm hatch into larvae within the small intestine. The larvae spread through the blood stream to different organs and finally arrive in the lung. From the lungs they eventually enter the throat and are swallowed. In the intestinal tract, the larvae complete development into adult worms. A female *A. lumbricoides* worm can produce 240,000 eggs daily, which pass within feces to the environment to begin the cycle anew. The eggs are resistant to cold weather and disinfectants and can remain viable for more than 10 years. Because of the high load of nematodes in ascariasis, there are also severe complications including intestinal obstruction, peritonitis, and acute pancreatitis [3]. There are different treatment strategies for ascariasis, such as surgery (in case of bowel obstruction) and application of anthelminthic drugs including albendazole, mebendazole, and pyrantel pamoate. At present and in the future, access to new broad-spectrum anthelmintics against *A. lumbricoides*, as well as hookworms and whipworms, are needed in countries where these infections are endemic [4].

Carbonic anhydrases (CAs) have been recently identified as potential targets for novel anti-infective drugs. CAs are encoded by six evolutionary divergent gene families: α, β, γ, δ, ζ, and η CAs [5–7]. All members of these gene families are metalloenzymes, which usually contain zinc ion in their catalytic active site [8]. Certain ζ- and γ-CAs contain cadmium (II), iron (II) or cobalt (II) as alternative metal ion cofactors [9–11]. α-CAs are the most intensively studied family, which contains 13 catalytically active members in mammals: cytosolic enzymes (CA I, CA II, CA III, CA VII, and CA XIII), membrane-bound (CA IV, CA IX, CA XII, CA XIV, and CA XV), mitochondrial CAs (VA and CA VB), and secreted CA (VI) [12]. β-CAs are found in plants, algae, fungi, bacteria, protozoans, arthropods, and nematodes [6, 13, 14]; γ-CAs in algae, plants, bacteria, and archaea [15]; δ-CAs in free-living marine dinoflagellates [16]; ζ-CAs in marine diatoms [9]; and η-CAs in *Plasmodium* parasites [5]. CAs play a critical role in many biochemical pathways, including respiration, pH homeostasis, electrolyte transfer, bone resorption, calcification, gluconeogenesis, lipogenesis, and ureagenesis [12, 17]. Because *β-CA* genes are absent in vertebrate genomes, while present in many parasite genomes, they are considered potential candidate target enzymes for novel anti-infectives [6, 7, 18, 19]. Literature on CA inhibition reveals that many inhibitors, such as sulfonamide, sulfamides, sulfamates, anions, phenols, coumarins, dithiocarbamates, fullerenes, boronic acids, carboxylates, polyamines, benzamides, hydroxymates, and mercaptans have been tested against β-CAs to control infectious organisms, such as *Candida albicans*, *Cryptococcus neoformans*, *Leishmania donovani, Salmonella typhimurium*, *Porphyromonas gingivalis*, *Helicobacter pylori*, *Streptococcus pneumoniae*, *Mycobacterium tuberculosis*, and *Brucella suis* [20–27]. Meanwhile, inhibitory studies have been also carried out on β-CAs from non-pathogenic model organisms, including *Saccharomyces cerevisiae* and *Drosophila melanogaster* [12, 28–34].

In this study, we analyzed properties of *A. lumbricoides* β-CA (AlBCA) using bioinformatics tools, produced AlBCA as a recombinant protein in insect cells, and tested its kinetic and inhibition properties. These investigations represent the first experimental study on a β-CA protein from a parasitic nematode.

## Methods

### Identification of AlBCA protein sequence

A β-CA protein sequence from *Caenorhabditis elegans* (Uniprot ID: Q2YS41) [35] was used for the initial NCBI BLAST protein homology search (http://blast.ncbi.nlm.nih.gov/Blast.cgi). Ten nematode β-CA protein sequences, including AlBCA, were aligned with the Clustal Omega algorithm to create a multiple sequence alignment (MSA) within the Jalview program (version 2.8.ob1) (http://www.jalview.org/).

### Structural and functional predictions based on AlBCA sequence

The AlBCA protein sequence (Uniprot ID: F1LE18) was used as a query in the integrative protein signature database, InterPro (http://www.ebi.ac.uk/interpro/). This database integrates together predictive models of representative protein domains, families, and functional sites from multiple and diverse databases, such as Gene3D, PANTHER, Pfam, PIRSF, PRINTS, ProDom, PROSITE, SMART, SUPERFAMILY, and TIGRFAMs [36]. The resulting InterPro ID (IPR001765) for AlBCA protein sequence was used as a query in the CATH/Gene3D database (http://www.cathdb.info/) [37]. This database hierarchically classifies domains into sequence and structure-based families and fold groups, when there is a sufficient evidence for having diverged from a common ancestor. The CATH/Gene3D database generated a rainbow model for superimposed AlBCA protein sequence and several other close species. We also identified the biochemical pathways and interactions of AlBCA through KEGG (Kyoto Encyclopedia of Genes and Genomes) (http://www.kegg.jp/) [38] and METACYC metabolic pathway databases (http://metacyc.org/) [39], which have both been linked to InterPro.

### Production of recombinant AlBCA

The CDS sequence of *AlBCA* gene was retrieved from the EMBL database (http://www.ebi.ac.uk/). GeneArt® gene synthesis technology (Life Technologies) was used to construct the *β-CA* gene sequence for insertion into the cloning vector (pFastBac1) [40]. DH10Bac cells (which contain the bacmid baculovirus shuttle vector, and a helper plasmid that produces the proteins needed for transposition), were transformed by pFast-*AlBCA* [7]. Then pFast-*AlBCA* was purified by PureLink™ HiPure Plasmid Purification Kit (Invitrogen).

Primary transfection of *Spodoptera frugiperda* (sf-9) insect cells for production of *Baculoviruses* was performed by HilyMax transfection reagent (Dojindo) (0.5×10^7^ cells/ml in 6-well plates), and the cells were incubated for 3 days at 29 °C. Cultured cells were centrifuged, passed through a 0.2 μm filter, and stored in a dark tube at +4 °C. For secondary transfection, 20 ml of cultured cells (2×10^6^/ml) were transfected with a primary stock of *Baculoviruses* and incubated for 3 days in a 29 °C shaker. Cultured cells were centrifuged, passed through 0.2 μm filter, and stored in a dark tube at +4 °C. For the expression of recombinant AlBCA, the secondary stocks of *Baculoviruses* were used to infect sf-9 cells (2×10^6^/ml) (using the same procedure as described for the production of the secondary stock, but at higher volumes).

The sf-9 cell culture medium was centrifuged at 5000 RPM for 10 min at room temperature. The supernatant containing secreted AlBCA was diluted at a ratio of 1:5 by binding buffer (0.1 M Tris, 0.2 M Na_2_SO_4_, pH 8). Then Protino™ Ni-NTA Agarose (Macherey-Nagel) was added to the diluted culture medium and put on a magnetic stirrer with a low rotation speed (2 h at room temperature and overnight incubation at +4 °C without stirring). The culture medium was poured into the funnel filtration system and passed through Whatman® filter paper by vacuum. The flow-through was collected in a separate vial. The agarose was washed with the wash buffer (50 mM Na_2_HPO_4_, 0.5 M NaCl, 20 mM Imidazol, pH 8). Elution buffer (50 mM Na_2_HPO_4_, 0.5 M NaCl, 0.25 M Imidazol, pH 8) was added into the column to finally elute the recombinant AlBCA. Thrombin treatment did not cleave the polyhistidine tag in spite of several attempts (data not shown) probably due to the specific molecular folding of AlBCA that completely hid the thrombin cleaving site. Therefore, the kinetic measurements were carried out using recombinant AlBCA containing the polyhistidine tag.

### Kinetic characterization of AlBCA

An Applied Photophysics stopped-flow instrument has been used for assaying the CA catalyzed CO_2_ hydration activity [41]. Phenol red (at a concentration of 0.2 mM) has been used as indicator, working at the absorbance maximum of 557 nm, with 20 mM TRIS (pH 8.3) as buffer, and 20 mM NaClO_4_ (for maintaining a constant ionic strength; this anion is not inhibitory against AlBCA up to concentrations of 50 mM, data not shown), following the initial rates of the CA-catalyzed CO_2_ hydration reaction for a period of 10–100 s. The CO_2_ concentrations ranged from 1.7 to 17 mM for the determination of the kinetic parameters (by Lineweaver-Burk plots) and inhibition constants. For each measurement at least six traces of the initial 5-10 % of the reaction have been used for determining the initial velocity. The uncatalyzed rates were determined in the same manner and subtracted from the total observed rates. The inhibition constant of acetazolamide (AAZ, 5-acetamido-1,3,4-thiadiazole-2-sulfonamide) was obtained by non-linear least-squares methods using PRISM 3 and the Cheng-Prusoff equation, as reported earlier [42, 43], and represents the mean from at least three different determinations.

## Results

### Identification of AlBCA protein sequence

**Fig. 1:**
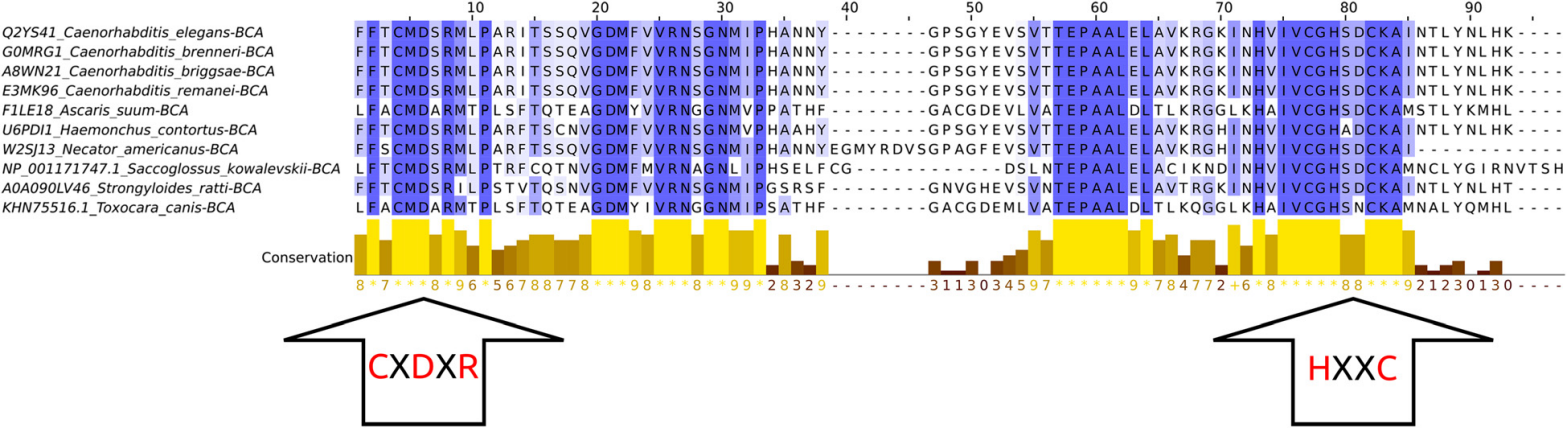
Multiple sequence alignment (MSA) of β-CA protein sequences from 10 nematodes. Only the central region of the sequences (85 amino acids, starting 3 amino acids prior to first highly conserved motif) are shown in this alignment. MSA revealed that all of them including AlBCA contain the first (CXDXR; C: cysteine, D: aspartic acid, R: arginine, X: any residue) and second (HXXC; H: histidine, C: cysteine, X: any residue) highly conserved sequences, which have been indicated by two black arrows at the bottom. Zinc ion (Zn^2+^) is the metal cofactor in catalytic active site of β-CAs, which binds to Cys from the first motif and His and Cys from the second motif [55]

Multiple sequence alignment (MSA) revealed that all 10 β-CA protein sequences from nematodes contained the first (CXDXR; C: cysteine, D: aspartic acid, R: arginine, X: any residue) and second (HXXC; H: histidine, C: cysteine, X: any residue) highly conserved sequence motifs of the catalytic site, which are the hallmark residues for β-CAs (Table 1, Fig. 1). In addition, the neighbor residues present within or close to the active site were almost identical.

**Table 1:** IDs for ten β-CA protein sequences from nematodes

Nematode name	β-CA protein IDs^a^
*Ascaris lumbricoides*	F1LE18
*Caenorhabditis brenneri*	G0MRG1
*Caenorhabditis briggsae*	A8WN21
*Caenorhabditis elegans*	Q22460 (bca-1)
	Q2YS41 (bca-2, isoform c)
	D3NQA9 (bca-2, isoform d)
*Caenorhabditis remanei*	E3MK96
*Haemonchus contortus*	U6PDI1
*Necator americanus*	W2SJ13
*Saccoglossus kowalevskii*	NP_001171747.1^b^
*Strongyloides ratti*	A0A090LV46
*Toxocara canis*	KHN75516.1

^a^Uniprot β-CA protein IDs

^b^NCBI β-CA protein ID

### Structural and functional predictions based on AlBCA sequence

**Fig. 2:**
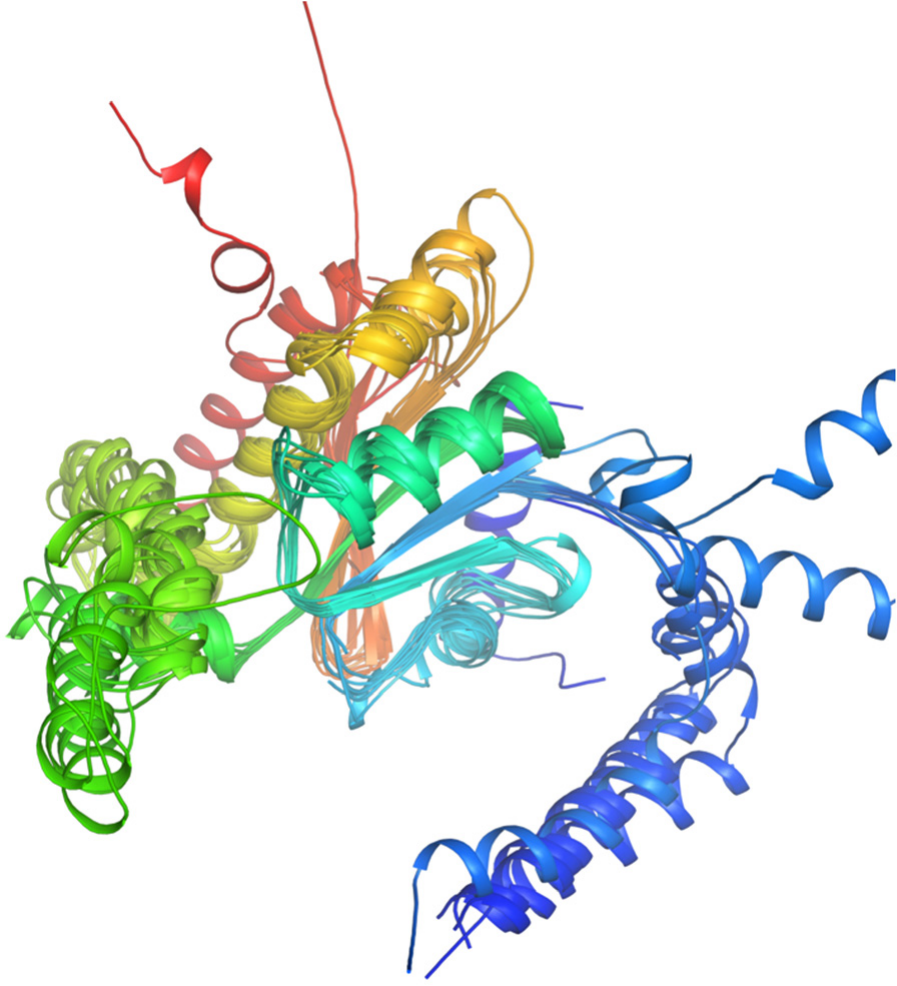
A rainbow model for superimposition of AIBCA and β-CAs of close relative species. This image has been generated from a superposition of nine representative domains within this superfamily. The domain positions and protein structures appear to be highly similar in this model

**Fig. 3:**
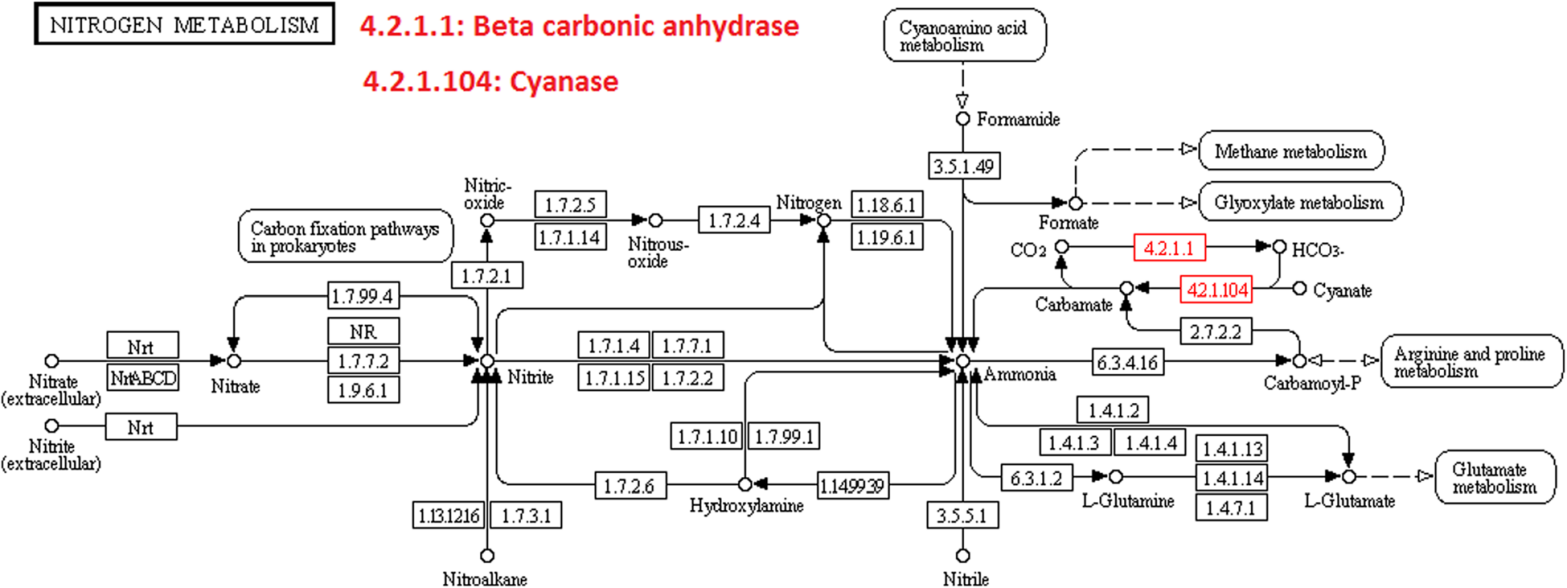
The predicted role of AlBCA in nitrogen metabolism pathway. This pathway has been generated by the KEGG (Kyoto Encyclopedia of Genes and Genomes) database. According to this model AlBCA catalyzes the conversion of CO_2_ and H_2_O to HCO_3_^−^. Cyanase converts cyanate to CO and NH in a bicarbonate-dependent reaction

**Fig. 4:**
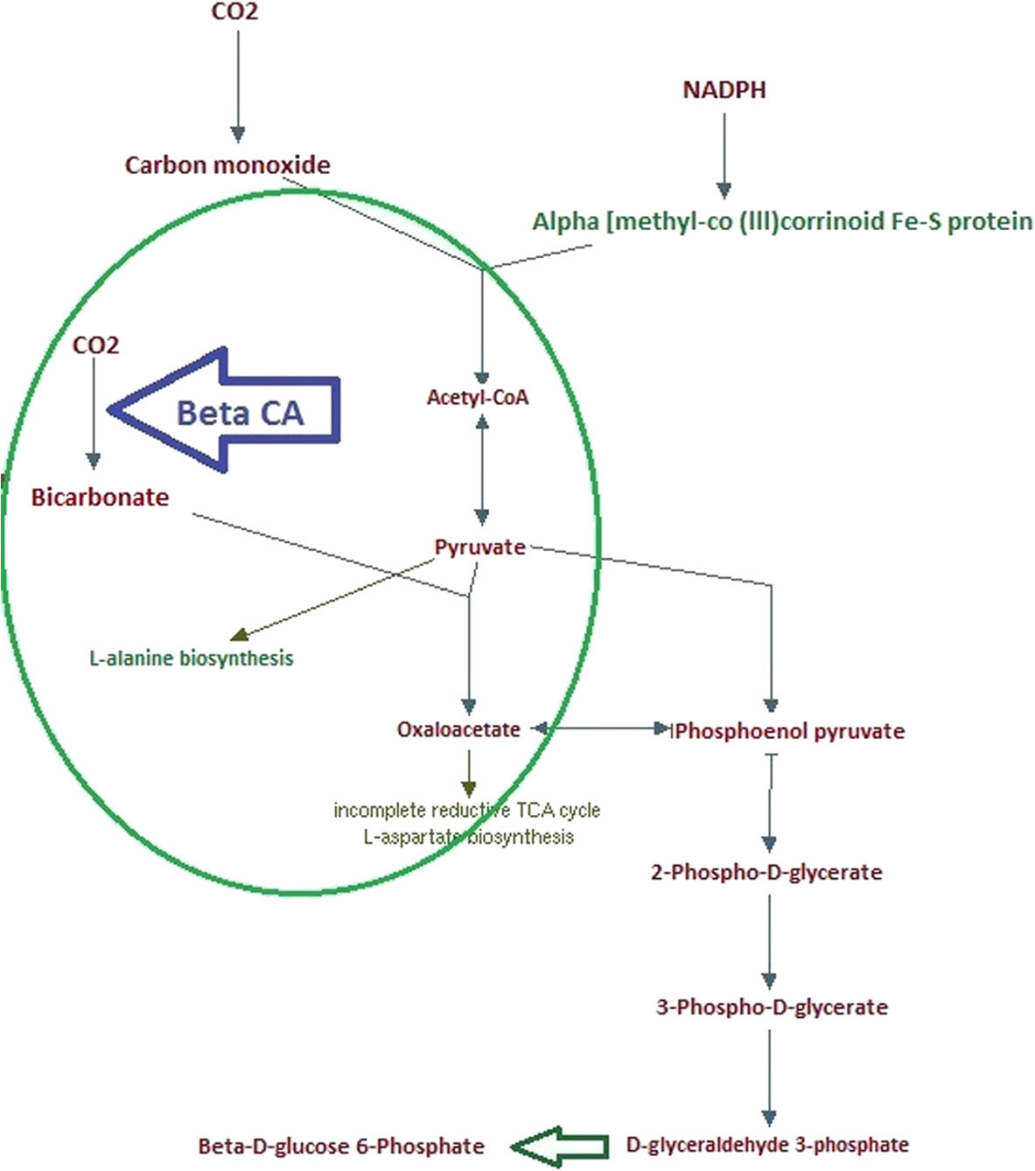
Role of AlBCA in gluconeogenesis ll pathway. The conversion of CO_2_ to HCO_3_^−^ is catalyzed by β-CA, which is shown with a blue arrow. Bicarbonate is then used for conversion of pyruvate to oxaloacetate

**Fig. 5:**
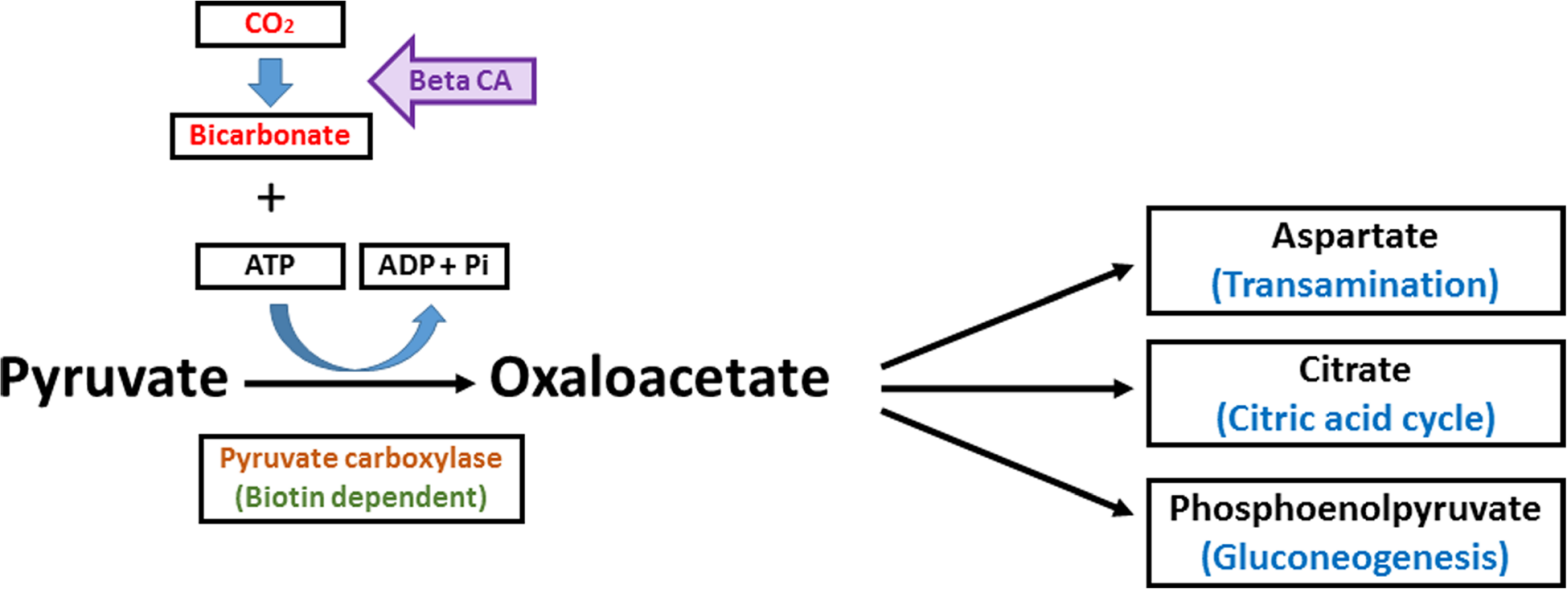
Role of bicarbonate generated by AIBCA in gluconeogenesis ll pathway. The conversion of CO_2_ to HCO_3_^−^ is catalyzed by β-CA, which is shown with a purple arrow. HCO_3_^−^ is a major element in conversion of pyruvate to oxaloacetate. Oxaloacetate is used to generate aspartate, citrate, and phosphoenolpyruvate through transamination, citric acid cycle, and gluconeogenesis pathways, respectively

Analysis of the AlBCA protein sequence by the InterPro database resulted in classification as part of the carbonic anhydrase family InterPro ID IPR001765. In the CATH/Gene3D database, AlBCA is categorized with the CATH superfamily ID 3.40.1050.10 (Beta-carbonic Anhydrase; Chain A). The tools of CATH/Gene3D database were used to generate a rainbow model for superimposition of AlBCA protein with other close relative β-CA proteins (Fig. 2). Metabolic pathway analyses of the AlBCA protein sequence in the KEGG and METACYC databases predict that the enzyme plays a major role in nitrogen metabolism (Fig. 3) and gluconeogenesis ll pathways (Figs. 4 and 5). Results from the KEGG database suggested that AlBCA might functionally participate in detoxification of cyanate by providing bicarbonate for cyanase enzyme. The METACYC database also predicted bicarbonate as the final product of the β-CA catalytic reaction. This bicarbonate would be needed for the mitochondrial gluconeogenic pathway where pyruvate is converted to oxaloacetate.

### Production and characterization of recombinant AlBCA

**Fig. 6:**
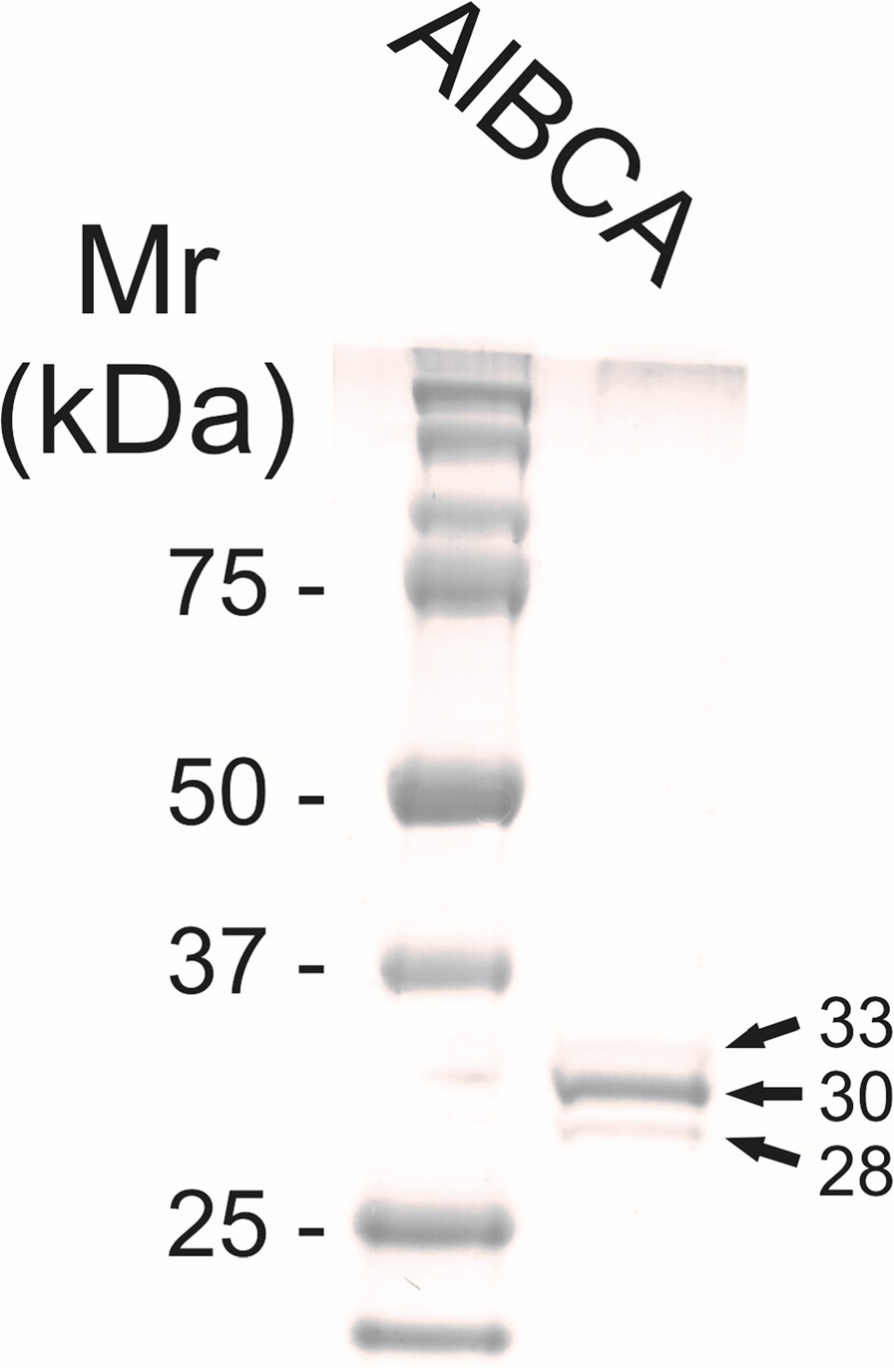
SDS-PAGE of AIBCA. Left lane shows the protein standard. The purified recombinant AIBCA appeared as a triple band (28, 30, and 33 kDa). The predicted molecular mass of His-tagged AlBCA was 30 kDa

A *β-CA* gene insert, including a thrombin cleavage site and a segment encoding a poly-histidine tag, was produced by GeneArt® technology and integrated into a pFastBac1 plasmid (Additional file 1: Figure S1). The purified plasmid was used to produce recombinant AlBCA in sf-9 cells. SDS-PAGE containing the eluted fraction showed three polypeptide bands of 28, 30, and 33 kDa. The 30-kDa band was the major product, which corresponds to the calculated molecular mass of AlBCA (Fig. 6).

### Kinetic properties of AlBCA

We have measured the kinetic properties of AlBCA for the physiologic reaction of CO_2_ hydration to bicarbonate and protons at pH 8.3 where, irrespective if this protein is a type I or type II β-CA, the active site should be open. The Zn (II) ion is coordinated by two Cys and one His residues as well as by a water molecule/hydroxide ion, acting as a nucleophile in the catalyzed reaction. We compared the activity of the new enzyme with those of well characterized α-class (human CA I and II) and β-CAs from *Leishmania donovani chagasi* (LdcCA), *Drosophila melanogaster* (DmBCA), and *Anopheles gambiae* (AgaCA), characterized earlier by our group [7, 21, 44]. As displayed in Table 2, AlBCA has significant catalytic activity in the physiologic reaction with the following kinetic parameters: k_*cat*_ of 6.0 × 10^5^ s^−1^ and k_*cat*_/K_*m*_ of 4.3 × 10^7^ M^−1^ s^−1^. The measured kinetic parameters show that AlBCA has a moderate activity, comparable to that of the relatively slower human carbonic anhydrase isoform hCA I, and in the same range as the enzymes characterized in *Anopheles gambiae* and *Leishamania donovani chagasi* [7, 21]. Furthermore, this activity has been effectively inhibited by the sulfonamide compound acetazolamide, which showed an inhibition constant of 84.1 nM. Although this was the only inhibitor investigated so far for this enzyme, work is in progress to understand in detail the inhibitory profiles of the main classes of activity modulators against this enzyme, i.e., anions, sulfonamides and their isosteres, and dithiocarbamates and their isosteres.

**Table 2:** Kinetic parameters for the CO_2_ hydration reaction catalysed by the human isozymes hCA I, II, III, IV, VA, VB, VI, VII, IX, XII, XIII, and XIV (α-class CAs) and the β-CAs from *Leishmania donovani chagasi* (LdcCA), *Drosophila melanogaster* (DmBCA), *Anopheles gambiae* (AgaCA) and *Ascaris lumbricoides* (AlBCA)

Enzyme	Class	kcat (s^−1^)	kcat/Km (M^−1^x s^−1^)	Ki (acetazolamide) (nM)^a^
hCA I	α	2.0×10^5^	5.0×10^7^	250 ± 12
hCA II	α	1.4×10^6^	1.5×10^8^	12 ± 0.8
hCA III	α	1.3×10^4^	2.5×10^5^	240000 ± 25000
hCA IV	α	1.1×10^6^	5.1×10^7^	74 ± 5.5
hCA VA	α	2.9×10^5^	2.9×10^7^	63 ± 2.1
hCA VB	α	9.5×10^5^	9.8×10^7^	54 ± 3.0
hCA VI	α	3.4×10^5^	4.9×10^7^	11 ± 0.7
hCA VII	α	9.5×10^5^	8.3×10^7^	2.5 ± 0.11
hCA IX	α	1.1×10^6^	1.5×10^8^	16 ± 0.8
hCA XII	α	4.2×10^5^	3.5×10^7^	5.7 ± 0.04
hCA XIII	α	1.5×10^5^	1.1×10^7^	16 ± 0.3
hCA XIV	α	3.1×10^5^	3.9×10^7^	41 ± 2.2
LdcCA	β	9.35×10^5^	5.9 ×10^7^	91.7 ± 5.7
DmBCA	β	9.5×10^5^	1.1×10^8^	516 ± 24
AgaCA	β	7.2×10^5^	5.6×10^7^	27.3 ± 2.0
AlBCA^a^	β	(6.0 ± 0.1) ×10^5^	(4.3 ± 0.2) ×10^7^	84.1 ± 2.9

^a^Mean ± standard error from three different assays

## Discussion

Multiple sequence alignment (MSA) has confirmed the presence of a β-CA enzyme in the proteome of *A. lumbricoides*, an important pathogen which is the causative agent of the parasitic roundworm disease, ascariasis. After this discovery our aim was focused on investigation of the putative functions of this enzyme by bioinformatics prediction tools, production as a recombinant protein, and characterization of its kinetic properties. We determined that AlBCA protein contains the first (CXDXR) and second (HXXC) hallmark sequences of β-CAs, and most of the neighbor residues present within or near the active site of the enzyme were also highly homologous compared to other aligned nine helminthic β-CAs. This result suggested that AlBCA is a catalytically active enzyme. The rainbow model of AlBCA protein was generated by superimposing its nine protein domains with β-CA proteins from close species. α-helices and β-sheets structures showed a high similarity in general domain architecture. We successfully produced recombinant AlBCA protein in Sf-9 insect cells. The SDS-PAGE analysis of the purified recombinant protein showed three polypeptide bands with molecular masses of 28, 30, and 33 kDa. Among them the middle band was clearly strongest and probably represents mature recombinant AlBCA protein. The lower 28-kDa polypeptide band variably appeared in SDS-PAGE analyses, and it probably represents a partially degraded protein. The upper 33-kDa polypeptide might represent a premature form of the enzyme. Even though our previous predictions for subcellular localization did not provide any definitive result [6], the AlBCA protein is probably either a mitochondrial or secretory protein like the other parasite β-CAs defined so far. Our previous studies have shown that there are examples of metazoan β-CAs in both subcellular locations [6]. Several species, such as *Saccoglossus kowalevskii, Trichinella spiralis,* and *Strigamia maritima*, possess mitochondrial β-CAs*.* The highest score for a secretory signal peptide was predicted to the β-CA of malaria mosquito *Anopheles darlingi*. In our previous analysis using the SignalP server, the β-CA of malaria mosquito *Anopheles darlingi* had the highest score in likelihood to be a secreted protein [6]. In the functional predictions performed by computational tools, AlBCA associated to conversion of CO_2_ and H_2_O to bicarbonate, as expected. Then bicarbonate was functionally linked to detoxification of cyanate, which is a toxic byproduct of some metabolites, such as urea and carbamoylphosphate. Cyanase catalyzes the decomposition of cyanate into CO_2_ and ammonia. Bicarbonate serves as a nucleophilic reactant that attacks and breaks down the cyanate, with carbamate as an unstable intermediate. Therefore, the role of CA in recycling of CO_2_ into bicarbonate, and the importance of bicarbonate in the nitrogen metabolism pathway whole metabolic process, are evident [45]. Furthermore, it was predicted that β-CA plays a role in gluconeogenesis ll pathway. If AlBCA locates in mitochondria, it would generate bicarbonate as the key element for conversion of pyruvate to other final products, such as aspartate, citrate, and phosphoenolpyruvate through transamination, citric acid cycle, and gluconeogenesis, respectively. Indeed, our results have indicated that AlBCA shows a significant catalytic activity for the conversion of CO_2_ into bicarbonate, as demonstrated *in vitro* by stopped-flow kinetic measurements.

Identification of β-CA from *C. elegans* in the Ensembl Metazoa database (http://metazoa.ensembl.org/index.html) [46] revealed that this model nematode contains three full-length β-CA protein sequences (Additional file 2: Table S1). An MSA of these β-CAs with AlBCA sequence, created with the Clustal Omega algorithm (http://www.ebi.ac.uk/Tools/msa/clustalo/) [47], showed that β-CA2 (isoform c, Uniprot ID: Q2YS41) from *C. elegans* is most similar to AlBCA. Thus, there is the possibility that the expression pattern of AlBCA is similar to *C. elegans* β-CA2 (isoform c). The expression of *C. elegans* β-CA2 (isoform c, Ensembl gene ID WBGene00013805) in the WormViz expression database (http://www.vanderbilt.edu/wormdoc/wormmap/WormViz.html) of WormBase (https://www.wormbase.org/#01-23-6) [48] showed that β-CA2 (isoform c) is expressed in all larval (EE, LE, L1, L2, L3, L4) and adult (including male and hermaphrodites) stages. The highest expression levels were detected in the body wall muscles of L2 stage. The results defined that β-CA is also present in larval neurons, muscles, coelomocytes, hypodermis layer, intestine, and excretory cells. In addition, β-CA2 (isoform c) is detectable in the whole body of adult male and hermaphrodite gonads of *C. elegans*. Previously, Fasseas *et al*. investigated the function of *C. elegans* β-CA using an RNAi technique [35]. They did not find any significant phenotypic change, which might be due to several reasons. First, other CA isoforms might compensate the lack of one β-CA. Second, the efficiency of gene silencing might have been inadequate due to challenges with RNAi technique. In another model organism, *D. melanogaster*, the highest upregulated values of β-CA mRNA were observed in the spermatheca (female), fat body, and heart of adults; as well as early larval stages and late in metamorphosis [7]. The knockdown studies of *D. melanogaster* β-CA showed a clear phenotypic change. Surprisingly, females were sterile and unable to produce eggs. An apparent defect was shown in migration of border cells, which probably contributed to infertility of female *D. melanogaster* [49].

For *in vivo* inhibitory studies, acetazolamide has been tested on live *C. elegans* [50] and *Plasmodium falciparum* (malaria parasite) [51]. These studies showed, however, that acetazolamide could not penetrate through the nematode cuticle or protozoan surface. A BLAST search using 14 human α-CAs as queries recently identified six α-CAs (CAH-1 to 6) in *C. elegans* [52]. Güzel *et al.* [53] carried out an inhibition study on *C. elegans* CAH-4 and 13 mammalian CAs using different sulfonamide derivatives including 2-(hydrazinocarbonyl)-3-phenyl-1H-indole-5-sulfonamides of type 1 and 2, and compared their inhibition efficacy with traditional CA inhibitors including acetazolamide (AZA) and ethoxzolamide (EZA). Their study defined that some of the new derivatives displayed excellent inhibitory action on CAH-4 with K_i_ as low as 6 nM, which was 5.83 times better value compared to AZA. Within this context, AZA and EZA showed moderate inhibitory effects on CAH-4. In another study, Giacomotto *et al.* [52] realized that *cah-4* null mutant *C. elegans* nematodes were nonviable, and knockdown experiments, using the RNAi technique, showed reduced muscle degeneration in dystrophin deficient muscle. Furthermore, *cah-4* RNAi treatment caused a significant shift in the dose response curves of CA inhibitors, methazolamide and dichlorphenamide. Giacomotto and coworkers concluded that their studies confirmed the suitability of *C. elegans* as a model organism for screening, identifying and characterizing potential lead pharmacological agents [52].

Albendazole and Mebendazole are drugs quite well-tolerated when used against ascariasis and resistance has not yet been a major issue. Novel anthelmintic approaches are needed, however, because global healthcare will likely face these challenges at some point, since millions of people require treatment [2, 54]. Inhibition of AlBCA by single dose and broad-spectrum inhibitors, which are effective against various helminthic infections, would be a novel strategy for treatment of ascariasis. It could potentially disrupt the normal detoxification of cyanate, which would in turn increase the intracellular cyanate concentration to a toxic level, leading to the death of the parasite.

## Conclusions

β-CAs represent promising targets for novel anti-parasitic drug design. In the future, new broad-spectrum, and preferably single dose β-CA inhibitors, should be designed against AlBCA and corresponding enzymes of whipworms and hookworms. For the moment, the clinically used sulfonamide acetazolamide, the only inhibitor tested so far, showed a promising *in vitro* inhibitory power, with an inhibition constant of 84.1 nM on AlBCA. Acetazolamide’s inability to penetrate the nematode is an obvious problem. Therefore, further studies should be planned to improve the penetration efficacy of CA inhibitors through biological membranes and cuticles of worms. The new sulfonamide derivatives, which were recently shown to inhibit *C. elegans* α-CAs, could represent useful leads for design of novel compounds having higher efficiency, better penetration, and minimal side effects on human CAs.

## Additional files

Additional file 1: Figure S1. Construction of pFast-AlBCA cloning vector for production of recombinant AlBCA. The construct contained the restriction sites for EcoR1 and Xho1, thrombin-cutting sequence, and 6× His-tag sequences. (TIFF 102 kb) Additional file 2: Table S1. Full-length β-CA protein sequences from *Caenorhabditis elegans*. (DOC 28 kb)

## Abbreviations

EE: Early embryo
LE: Late Embryo
L1: Larval stage 1
L2: Larval stage 2
L3: Larval stage 3
L4: Larval stage 4
