# Supplementary figures and images for: Ascaris lumbricoides β carbonic anhydrase: a potential target enzyme for treatment of ascariasis
Source: Parasit Vectors. 2015 Sep 18;8:479. doi: 10.1186/s13071-015-1098-5 (PMC4575479; doi:10.1186/s13071-015-1098-5)

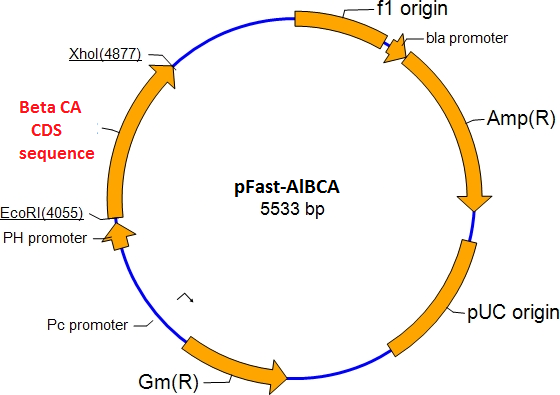

Supplement: Additional file 1: Figure S1. — Construction of pFast-AlBCA cloning vector for production of recombinant AlBCA. The construct contained the restriction sites for EcoR1 and Xho1, thrombin-cutting sequence, and 6× His-tag sequences. (TIFF 102 kb) [file 13071_2015_1098_MOESM1_ESM.tif]
