# Supplementary material for: Ascaris lumbricoides β carbonic anhydrase: a potential target enzyme for treatment of ascariasis
Source: Parasit Vectors. 2015 Sep 18;8:479. doi: 10.1186/s13071-015-1098-5 (PMC4575479; doi:10.1186/s13071-015-1098-5)
Supplement: Additional file 2: Table S1. — Full-length β-CA protein sequences from Caenorhabditis elegans. (DOC 28 kb) [file 13071_2015_1098_MOESM2_ESM.doc]

**Supplementary data**

**Table S1. Full-length β-CA protein sequences from *Caenorhabditis elegans.***

| **Species name** | **Protein name** | **Gene name** | **Uniprot protein ID** | **Ensembl gene ID** | **Ensembl transcript ID** |
| --- | --- | --- | --- | --- | --- |
| *Caenorhabditis elegans* | Beta carbonic anhydrase 1  Beta carbonic anhydrase 2 (isoform c)  Beta carbonic anhydrase 2 (isoform d) | bca-1  bca-2  bca-2 | Q22460  Q2YS41  D3NQA9 | WBGene00000245  WBGene00013805  WBGene00013805 | T13C5.5  Y116A8C.28c  Y116A8C.28d |
